# A plasmon-enhanced theranostic nanoplatform for synergistic chemo-phototherapy of hypoxic tumors in the NIR-II window†

**DOI:** 10.1039/d1sc01760h

**Published:** 2021-07-13

**Authors:** Ming-Ming Chen, Hai-Li Hao, Wei Zhao, Xueli Zhao, Hong-Yuan Chen, Jing-Juan Xu

**Affiliations:** State Key Laboratory of Analytical Chemistry for Life Science, School of Chemistry and Chemical Engineering, Nanjing University Nanjing 210023 China weizhao@nju.edu.cn xujj@nju.edu.cn; Institute of Nanochemistry and Nanobiology, School of Environmental and Chemical Engineering, Shanghai University Shanghai 200444 China; College of Chemistry and Molecular Engineering, Zhengzhou University Zhengzhou 450001 China

## Abstract

Development of simple and effective synergistic therapy by combination of different therapeutic modalities within one single nanostructure is of great importance for cancer treatment. In this study, by integrating the anticancer drug DOX and plasmonic bimetal heterostructures into zeolitic imidazolate framework-8 (ZIF-8), a stimuli-responsive multifunctional nanoplatform, DOX-Pt-tipped Au@ZIF-8, has been successfully fabricated. Pt nanocrystals with catalase-like activity were selectively grown on the ends of the Au nanorods to form Pt-tipped Au NR heterostructures. Under single 1064 nm laser irradiation, compared with Au NRs and Pt-covered Au NRs, the Pt-tipped Au nanorods exhibit outstanding photothermal and photodynamic properties owing to more efficient plasmon-induced electron–hole separation. The heat generated by laser irradiation can enhance the catalytic activity of Pt and improve the O_2_ level to relieve tumor hypoxia. Meanwhile, the strong absorption in the NIR-II region and high-*Z* elements (Au, Pt) of the DOX-Pt-tipped Au@ZIF-8 provide the possibility for photothermal (PT) and computed tomography (CT) imaging. Both *in vitro* and *in vivo* experimental results illustrated that the DOX-Pt-tipped Au@ZIF-8 exhibits remarkably synergistic plasmon-enhanced chemo-phototherapy (PTT/PDT) and successfully inhibited tumor growth. Taken together, this work contributes to designing a rational theranostic nanoplatform for PT/CT imaging-guided synergistic chemo-phototherapy under single laser activation.

## Abstract 2

A plasmon-enhanced theranostic nanoplatform for synergistic chemo-phototherapy (PTT/PDT) of hypoxic tumors in the NIR-II window.

## Graphical abstract

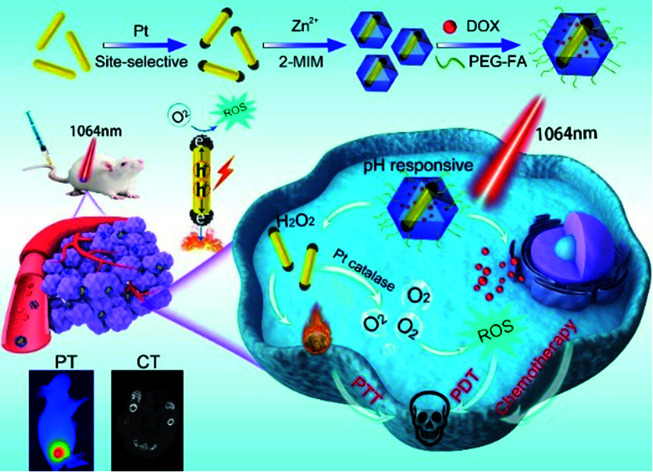

## Introduction

With the great development of diagnostic and theranostic nanomedicine, a variety of treatment modalities have been widely investigated in the field of cancer treatment.^1–4^ Chemotherapy, radiation therapy and surgery are still the main therapies in the clinical treatment of cancer; however, they are often limited by multidrug resistance (MDR), unsatisfactory therapeutic efficiency and side effects.^5–7^ Nowadays, light-triggered theranostic strategies like photothermal therapy (PTT) and photodynamic therapy (PDT) have drawn great attention due to their minimal invasiveness, high temporal and spatial controllability, and negligible side effects.^8–10^

Photothermal therapy (PTT) adopts photothermal conversion agents (PTAs) to harvest light energy to induce localized hyperthermia and kill cancer cells.^11^ Compared with PTAs which absorb near-infrared (NIR) light located at the NIR-I region (700–900 nm), PTAs with photothermal absorption in the NIR-II window (1000–1700 nm) have shown superior tumor therapeutic efficiency owing to their deeper tissue penetration and larger maximum permissible exposure.^12–16^ Photodynamic therapy (PDT) usually utilizes light to activate a photosensitizer to generate reactive oxygen species (ROS) in the presence of O_2_. Meanwhile, hypoxia acts as one of the phenotype features of the tumor microenvironment, which could lead to ineffective therapeutic effects of O_2_-dependent PDT.^17–20^ Besides, a combination of both PDT and PTT is usually required to integrate a distinct photosensitizer agent (PSA) and photothermal agent (PTA) in a single system with sequential irradiations by two different lasers, which results in a prolonged treatment duration and intricate treatment process.^21,22^ Therefore, it is highly desired to develop smart nanomaterials with excellent photodynamic and photothermal effects that could overcome hypoxia in a tumor microenvironment (TME) to effectively eliminate tumors upon single NIR-II laser irradiation.

Noble metal nanoparticles (NPs), such as Au, Pd, and Pt, have attracted extensive attention in the therapeutic field owing to their tunable localized surface plasmon resonance (LSPR) characteristics, good biocompatibility, strong NIR light absorption and enzyme-like activity across the visible and near-infrared spectral range.^23–28^ It is well known that the incident light on plasmonic metal nanoparticles could excite localized surface plasmon resonance (LSPR), which leads to strong enhancement of the electromagnetic field and energetic charge carrier generation.^29,30^ Importantly, the generated energetic charges can promote reactive oxygen species (ROS) generation by both chemical and energy transformation processes, and release the heat through a non-radiative transition process, endowing the plasmonic nanostructures with photodynamic and photothermal properties.^31,32^ Moreover, these plasmonic metal nanomaterials (Pt) could act as nanozymes with catalase-like activity, thereby catalyzing the production of O_2_ in H_2_O_2_ (100 μM to 1 mM) overexpressed in TME.^33–36^ Thus, anisotropic plasmonic metal heterostructures with enzyme-mimicking activities and excellent energetic charge carrier generation ability have great potential in cancer treatments.

Metal–organic frameworks (MOFs) self-assembled from metal ions or clusters and organic ligands through coordination bonds have attracted considerable attention in catalysis,^37^ separation^38^ and biomedical applications.^39^ Among them, zeolitic imidazolate framework-8 (ZIF-8), a typical MOF built from low-toxicity Zn^2+^ and 2-methylimidazole (2-MIM), has emerged as a potent platform in biomedical fields owing to its structural tailorability, excellent biocompatibility, feasible functionality and intrinsic biodegradability.^40^ A series of multifunctional nanocomposites based on ZIF-8 encapsulated with nanoparticles,^41^ molecules^42^ and enzymes^43^ have received much attention for theranostics. As a pH-responsive matrix for drug delivery, it is stable under physiological conditions while decomposing in the acidic tumor microenvironment.^44^ Hence, it is of great significance to rationally design an “all-in-one” nanoplatform that simultaneously integrates multiple kinds of functional nanoparticle and molecule in ZIF-8 for high therapeutic efficacy.

**Scheme 1:**
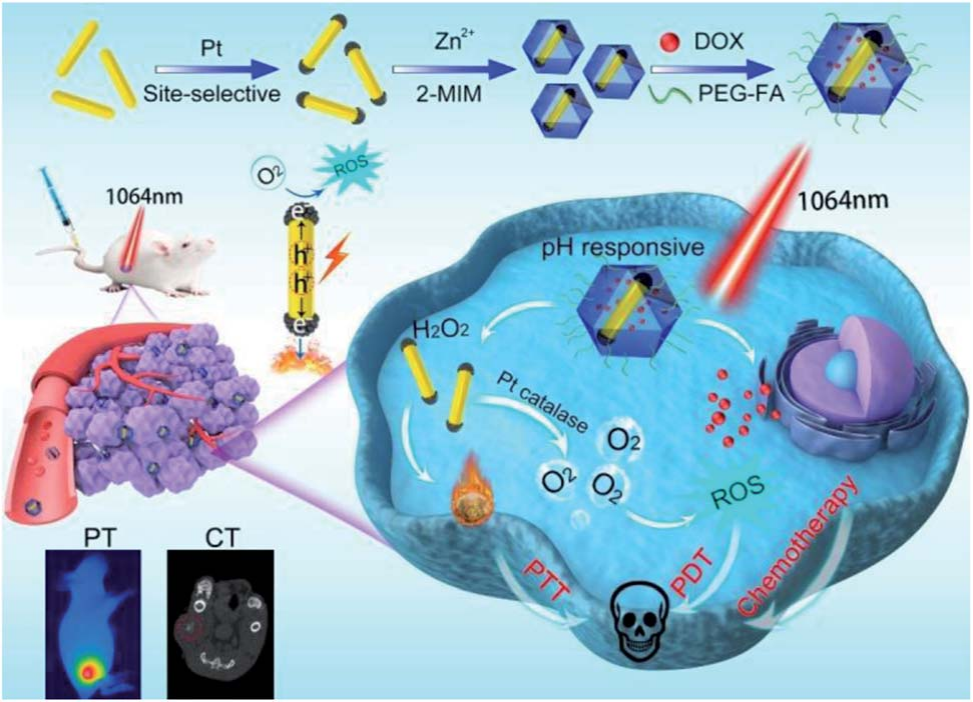
Schematic illustration for the fabrication of a DOX-Pt-tipped Au@ZIF-8 nanoplatform and the multimodal imaging guided synergistic anticancer therapy under a single NIR-II laser.

Herein, we have designed and fabricated a biocompatible multifunctional nanocomposite, DOX-Pt-tipped Au NRs@ZIF-8, for PT/CT imaging and synergistic chemo-phototherapy (Scheme 1). Pt dots were site-selectively grown through preferential adsorption at the two ends of Au NRs to form anisotropically plasmonic bimetal nanostructures (Pt-tipped Au NRs) with the LSPR maximum at the NIR-II window. Under single NIR-II laser (1064 nm) irradiation, compared with Au NRs and Pt dot covered Au NRs, more efficient electron–hole spatial separation could occur in Pt-tipped Au NRs. The generated hot electrons can release heat by non-radiative transition and produce ROS based on energy and chemical transformation processes, revealing excellent photothermal and photodynamic performance. Moreover, Pt exhibited intrinsic catalase-like activity which could catalyze endogenous H_2_O_2_ to continuously generate O_2_, which was even significantly enhanced under the NIR-II laser irradiation and thereby overcame tumor hypoxia. The ZIF-8 shell simultaneously encapsulated Pt-tipped Au NRs and DOX, which released these therapeutic agents stimulated by mild acidity in the tumor microenvironment. Meanwhile, due to the strong absorption at the NIR-II region and the high atomic number elements (Au, Pt), Pt-tipped Au NRs were a contrast agent for both *in vitro* and *in vivo* imaging including computed tomography (CT) and photothermal (PT) imaging. All in all, the DOX-Pt-tipped Au NRs@ZIF-8 exhibits great potential in bimodal imaging diagnosis and synergistic chemo-phototherapy (PTT/PDT) with remarkable tumor therapeutic efficacy.

## Results and discussion

### Characterization of the DOX-Pt-tipped Au@ZIF-8 nanocomposite

**Fig. 1:**
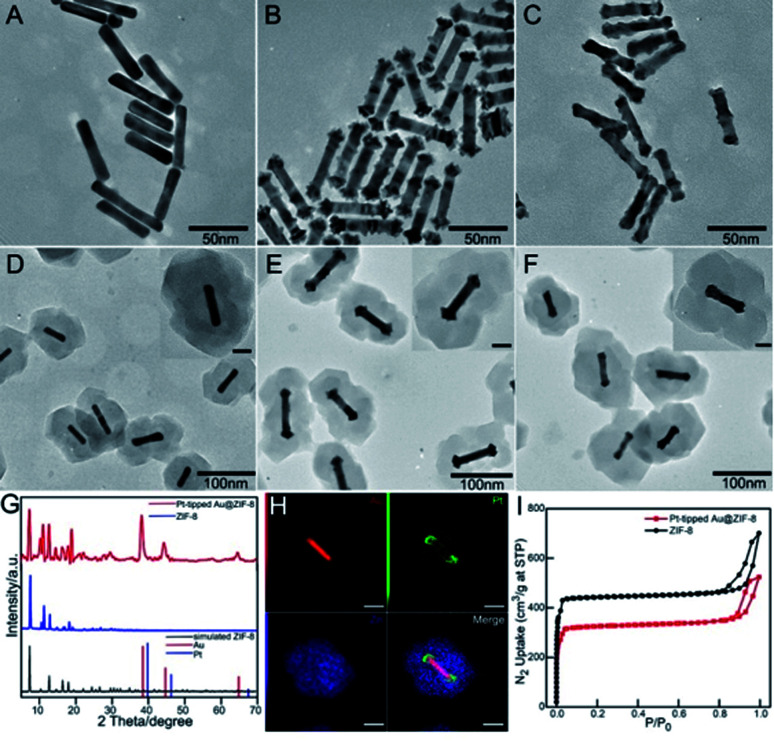
(A–C) TEM images of Au NRs, Pt-tipped Au NRs and Pt-covered Au NRs. TEM morphological characterization of (D–F) the corresponding Au@ZIF-8, Pt-tipped Au@ZIF-8 and Pt-covered Au@ZIF-8. Inset: the HR-TEM images. XRD patterns (G) and N_2_ adsorption/desorption isotherms (I) of pure ZIF-8 and Pt-tipped Au@ZIF-8. (H) STEM-EDX elemental mapping images of Au, Pt, Zn and Pt-tipped Au@ZIF-8.

The synthesis procedure of multifunctional DOX-Pt-tipped Au@ZIF-8 nanocomposites is illustrated in Scheme 1. Firstly, pre-grown Au nanorods with an average length and diameter of 48 nm and 9.2 nm (Fig. 1A) were prepared according to the classical seed-mediated method with some modification.^45^ Site-selective overgrowth of Pt on Au NRs was obtained through the adjustment of Ag^+^ ions in solution.^46^ As shown in Fig. 1B and C, in the absence of Ag^+^, Au NRs completely covered by Pt were formed and the presence of Ag^+^ led to preferential growth at both ends of the Au NRs. After tip-coating, the average length and diameter of Pt-tipped Au NRs (over 50 rods for analysis) were 58 nm and 10 nm with an average aspect ratio of 5.8. The representative transmission electron microscopy (TEM) images (Fig. 1D–F) revealed that the ZIF-8 shell was deposited onto the Au NRs, Pt-tipped Au and Pt-covered Au NRs with an average size of ∼150 nm. The characteristic X-ray diffraction (XRD) peaks of the ZIF-8 and Pt-tipped Au@ZIF-8 nanoparticles (Fig. 1G) were detected with good agreement with the standard patterns of the face-centered cubic Au, Pt and theoretical ZIF-8. The STEM-EDS elemental mapping (Fig. 1H) further showed that Pt was located only on the two ends of Au NRs while Zn of ZIF-8 was homogeneously assembled on the surface of the nanostructure. Besides, the elemental mapping of Pt-covered Au@ZIF-8 (Fig. S1†) revealed that the Pt dots were fully coated on the Au NRs. The N_2_ adsorption–desorption analysis of Pt-tipped Au@ZIF-8 presented a representative type I curve (Fig. 1I), indicating that the structure was mainly microporous.^47,48^ The pore size distribution of Pt-tipped Au@ZIF-8 (Fig. S2†) is composed of three species of micropores with diameters of 9.9, 12.7, and 15.9 Å, which is consistent with that of pristine ZIF-8. Owing to the special micro-porous structure of the ZIF-8 shell, the anticancer drug DOX could be loaded in Pt-tipped Au@ZIF-8 and mPEG-FA was modified on the surface of the ZIF-8 shell to obtain the final nanocomposites, which was verified by the zeta potential and dynamic light scattering (DLS) analysis (Fig. S3†). The loading efficiency of DOX in the Pt-tipped Au@ZIF-8 nanostructures was calculated to be 24.9% by measuring the absorption of DOX at 480 nm (Fig. S4†).

### Photothermal, catalase activity, photodynamic and drug release properties

**Fig. 2:**
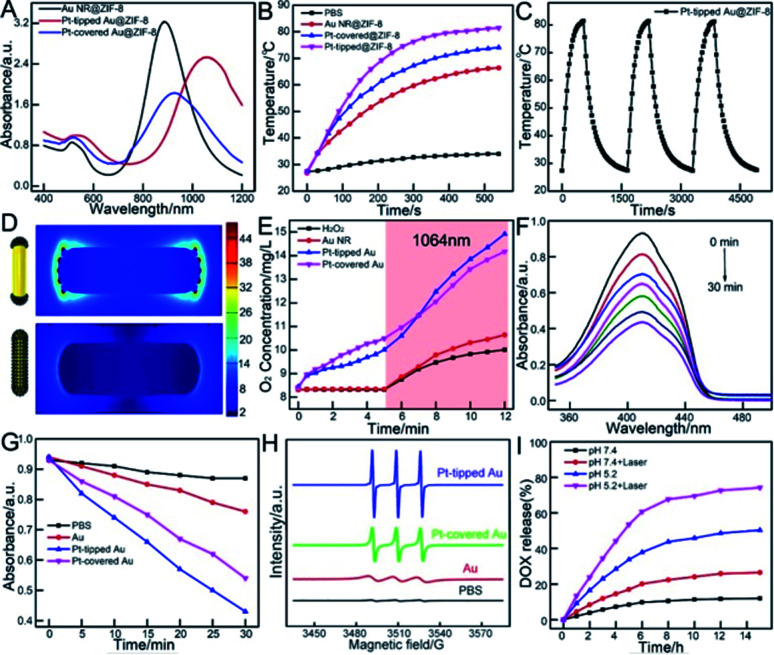
(A) UV-vis-NIR absorption spectra of Au@ZIF-8, Pt-tipped Au@ZIF-8 and Pt-covered Au@ZIF-8. (B) Temperature–time curves of different nanomaterials (100 μg mL^−1^) under 1064 nm laser irradiation (1 W cm^−2^). (C) The photothermal stability of the Pt-tipped Au@ZIF-8 for three successive cycles of on/off laser irradiation. (D) Electric field enhancement distributions at LSPR excitation based on FDTD simulation. (E) The O_2_ concentration of H_2_O_2_ incubated with different nanoparticles under 1064 nm laser irradiation from the 5th min to the 12th min. (F) Time dependent decrease in the UV-vis absorption spectra of DPBF treated with Pt-tipped Au NRs. (G) Decay curves of DPBF absorption at 410 nm with different nanomaterials. (H) ESR spectra of TEMP/^1^O_2_ adducts collected from H_2_O_2_ incubated with different nanoparticles. (I) DOX release profiles from DOX-Pt-tipped Au@ZIF-8 complexes with and without NIR laser irradiation at different pH values.

As the porous ZIF-8 shell has little influence on the position of the LSPR band of the Au NR, and Pt-tipped and Pt-covered Au NR core,^49^ the core nanostructures are primarily responsible for the photoabsorption behavior of core@ZIF-8 nanostructures (Fig. 2A). The UV-vis-NIR spectrum of Au NRs was centered at about 880 nm with an average aspect ratio of 5.2. For the Pt-tipped Au NR heterostructure, it shows a strong red shift to about 1060 nm in the NIR-II window because of the relative changes in the nanorod shape and the increase of aspect ratio after tip growth.

For Pt-covered Au NR core@shell nanostructures, an obvious decrease in LSPR intensity and slight red shifts were observed on account of the complete encapsulation of the Au NR core in the Pt shell and suppression of light absorbance.^50,51^ As displayed in Fig. 2B, under 1064 nm laser irradiation (1 W cm^−2^, 9 min), the Pt-tipped Au@ZIF-8 has superior photothermal activity compared with other nanostructures, which rapidly increased from 27.4 °C to 81.4 °C within 9 min due to the good wavelength match and strong absorption under 1064 nm laser irradiation. Pt-tipped Au@ZIF-8 could induce the most potent photothermal activity with a photothermal conversion efficiency of 42.1%, followed by Pt-covered Au@ZIF-8 (40.2%) and Au NR@ZIF-8 (39.2%) (calculation details in the ESI and Fig. S6†). In addition, the Pt-tipped Au@ZIF-8 exhibits good photothermal stability upon 1064 nm laser irradiation after three heating (each for 9 min) and cooling cycles (Fig. 2C). Fig. 2D and S5C† show the spatial distribution of the LSPR-induced enhancement of electric field intensity in Pt-tipped and Pt-covered Au NRs and longer Au NRs with an aspect ratio of 6.2 through 3D FDTD simulation. The more significant electric field enhancement occurred at the ends of Pt-tipped Au NRs, corresponding to more hot electron generation.^52^ It has been reported that nano-Pt possessed excellent catalase-like activity to induce the decomposition of H_2_O_2_ to O_2_.^53,54^ To verify this, the dissolved O_2_ production was monitored with a dissolved oxygen meter. As shown in Fig. 2E, in the first 5 min without laser irradiation the Pt-covered Au NRs produce more O_2_ than the Pt-tipped Au NRs owing to more Pt dot distribution. In contrast, no significant O_2_ generation was detected for PBS and Au NRs. Meanwhile, under 1064 nm laser irradiation the amount of O_2_ generated by bimetallic PtAu nanorods quickly increased. After 7 min irradiation, the Pt-tipped Au NR heterostructures possessed the best O_2_ generation capability, which demonstrated that the localized surface plasmon resonance (LSPR) effect of noble metals could effectively promote the catalytic reaction.^55^ Then, the ^1^O_2_ generation performance of Au NRs, and Pt-tipped and Pt-covered Au NRs was tested by using 1,3-diphenylisobenzofuran (DPBF) as an indicator. Fig. 2F indicates that the absorption peak intensity of DPBF at 410 nm was continuously decreased within 30 min when treated with Pt-tipped Au NRs under 1064 nm laser irradiation. Compared with other nanostructures, the heterostructures with Pt coated only on the ends have the strongest ROS generation ability owing to more efficient electron–hole separation under 1064 nm laser irradiation (Fig. 2G). Besides, electron spin resonance (ESR) spectroscopy (with TEMP as a trapping probe for ^1^O_2_) was used as direct evidence for the generated ROS, which also verified that the Pt-tipped Au NRs have the best ROS generation performance (Fig. 2H) owing to more O_2_ and hot electron generation. Then, the drug release behavior from the DOX-Pt-tipped Au@ZIF-8 nanocomposite was studied in solutions at different pH values with or without 1064 nm laser irradiation. As shown in Fig. 2I, due to pH-responsive dissociation of the Zn–O and Zn–N coordination bonds of the ZIF-8,^49^ the drug release in solution at pH 5.2 up to 50.3% was far faster than that in solution at pH 7.4 within 14 h. The drug release was also greatly affected by the laser irradiation. With 1064 nm laser irradiation the cumulative release rate of DOX was nearly 1.6 times higher than that without laser irradiation, which was attributed to the high temperature caused by the photothermal effect that accelerates the dissociation of Zn–O coordination. Correspondingly, the TEM images in Fig. S7† also confirmed the breaking and dissolution of the ZIF-8 nanostructures by low pH and laser irradiation.

### *In vitro* cytotoxicity and therapeutic effect of the nanoplatform

**Fig. 3:**
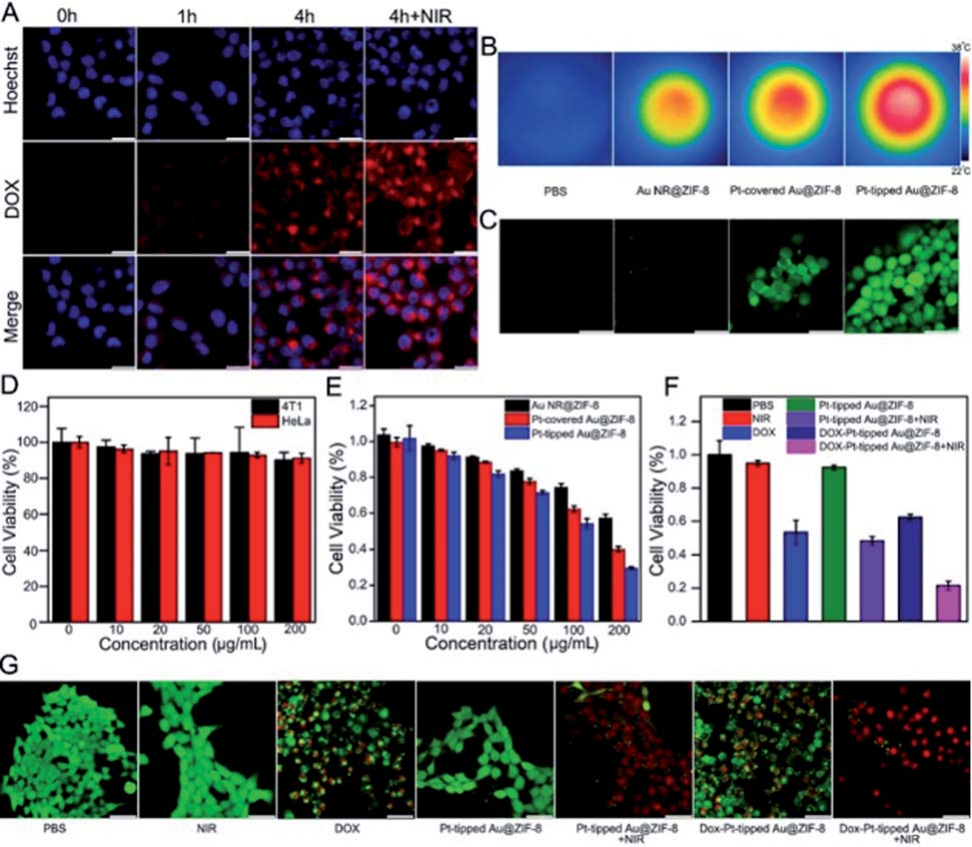
(A) Confocal images of 4T1 cell incubation with DOX-Pt-tipped Au@ZIF-8 (100 μg mL^−1^) at different times with or without 1064 nm laser irradiation. Scale bar: 25 μm. (B) Infrared thermal images of the Au@ZIF-8, Pt-tipped Au@ZIF-8 and Pt-covered Au@ZIF-8 aqueous solutions (100 μg mL^−1^) irradiated by a 1 W cm^−2^ 1064 nm laser for 5 min *in vitro*. (C) Fluorescence images of intracellular ROS generation from intact 4T1 cells incubated with different nanomaterials (100 μg mL^−1^). Scale bar: 25 μm. (D) The viability rate of 4T1 cells and HeLa cells incubated with varied concentrations of Pt-tipped Au@ZIF-8. (E) MTT viability assessment of 4T1 cells treated with different concentrations of Au@ZIF-8, Pt-covered Au@ZIF-8 and Pt-tipped Au@ZIF-8 for 24 h under 5 min of 1064 nm laser irradiation (1 W cm^−2^). (F) Cell viability of 4T1 cells with different treatments. (G) The fluorescence imaging of 4T1 cells stained by calcein AM (green) and PI (red) after different treatments. Scale bar: 25 μm.

To study the cellular uptake of the DOX-Pt-tipped Au@ZIF-8 nanocomposite (loaded with 25 μg mL^−1^ of DOX), the nanocomposite-treated 4T1 cells were observed and recorded by confocal laser scanning microscopy (CLSM). As shown in Fig. 3A, the cell nuclei were stained with Hoechst 33342 as blue fluorescence, the red fluorescence of DOX could be seen in the cells after incubating for 1 h, and intracellular accumulation of DOX increased with the extended coincubation time. The cellular uptake of nanoparticles was through cell endocytosis and mainly localized within the cytoplasmic region (Fig. S8†). Moreover, the 1064 nm laser irradiation could effectively enhance the fluorescence intensity of DOX, which indicated that the laser irradiation promoted the intracellular release of DOX. From Fig. 3B, obviously bright IR images of four sets of 4T1 cells cultured under different conditions were investigated. Compared with other nanostructures, the 4T1 cells incubated with the Pt-tipped Au@ZIF-8 with the same 1064 nm laser irradiation shows the highest temperature changes. The intracellular ROS generation efficiency of different nanostructure composites was evaluated with the nonfluorescent 2′,7′-dichlorofluorescein diacetate (DCFH-DA) in 4T1 cells, which could be rapidly oxidized by ROS to generate green fluorescence. As displayed in Fig. 3C, both the Pt-covered and Pt-tipped Au@ZIF-8 treated cells exhibited bright green fluorescence, implying efficient ROS production. Among them, the Pt-tipped Au@ZIF-8 nanostructures presented the brightest green fluorescence signals, confirming that the Pt induced preferential growth on the tips could overcome the hypoxic environment to generate ROS efficiently due to the greater generation of oxygen and electron–hole pairs during laser irradiation. The dose dependent cytotoxicity of the Pt-tipped Au@ZIF-8 nanocomposite was examined against HeLa and 4T1 cells by MTT assays. The cells were incubated with diverse concentrations (0, 10, 20, 50, 100, and 200 μg mL^−1^) for 24 h under dark conditions. The results in Fig. 3D indicated that the cell survival rate was still more than 90% and the Pt-tipped Au@ZIF-8 itself has good biocompatibility, even for normal cells (Fig. S9†). In addition, we compared the cell viability of different structural nanocomposites at various concentrations under 1064 nm laser irradiation (Fig. 3E). It is obviously seen that the cell killing ability of Pt-tipped Au@ZIF-8 is far superior to those of Au NR@ZIF-8 and Pt-covered Au@ZIF-8, which was attributed to the excellent photothermal performance and greater ROS generation. Furthermore, the cell survival rates against DOX, Pt-tipped Au@ZIF-8 and DOX-Pt-tipped Au@ZIF-8 with and without 1064 nm laser irradiation are displayed in Fig. 3F. The control group, the laser group and the only Pt-tipped Au@ZIF-8 group show no obvious cell apoptosis. In contrast, the cell viability rate of the Pt-tipped Au@ZIF-8 with the laser irradiation group decreased to 48.5% due to the NIR-II laser induced photothermal therapy (PTT) and photodynamic therapy (PDT). The cells treated with DOX and DOX-Pt-tipped Au@ZIF-8 showed cytotoxic effects to a certain extent owing to the toxicity of the drug DOX itself. It is noteworthy that the DOX-Pt-tipped Au@ZIF-8 with 1064 nm laser irradiation combining synergistic chemotherapy, photothermal therapy and photodynamic therapy exhibited a significantly enhanced anticancer effect with the cell viability dramatically decreasing to 21.5%. Additionally, the live and dead cell double-staining by calcein AM (green) and PI (red) was carried out to study the cell killing efficiency under different treatment conditions (Fig. 3G). With 1064 nm laser irradiation, the cells cultured with DOX-Pt-tipped Au@ZIF-8 were completely destroyed with red fluorescence due to the enhanced synergistic therapeutic effect, which is also consistent with the MTT assay.

### *In vivo* thermal imaging and CT imaging

**Fig. 4:**
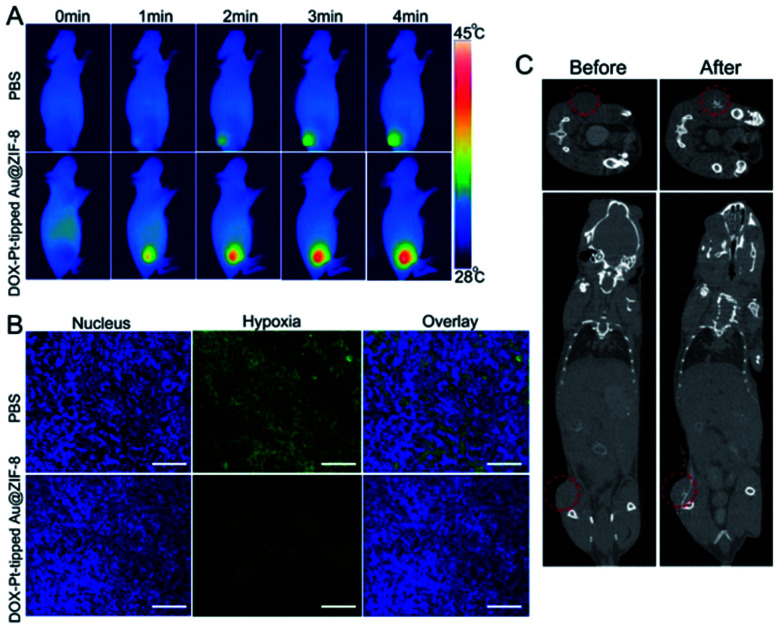
(A) *In vivo* infrared thermal images of 4T1 tumor-bearing mice after injection of either PBS or DOX-Pt-tipped Au@ZIF-8 with different irradiation times under NIR laser irradiation at 1064 nm (1 W cm^−2^). (B) HIF-1a staining of 4T1 tumor treated with PBS and DOX-Pt-tipped Au@ZIF-8 + NIR-II laser irradiation, respectively (blue color indicates cell nucleus; green color indicates HIF-1a). Scale bar: 50 μm. (C) Top: CT images of tumor tissues of mice before and after intratumoral injection of DOX-Pt-tipped Au@ZIF-8 (10 mg kg^−1^). Bottom: 3D reconstructed CT images of tumor-bearing mice before and after intratumoral injection of DOX-Pt-tipped Au@ZIF-8 (10 mg kg^−1^).

Encouraged by the excellent anticancer results *in vitro*, mice bearing 4T1 tumors were chosen as models for evaluating the *in vivo* therapeutic efficacy. Based on the good photothermal conversion efficiency in the NIR-II region, the photothermal properties of the DOX-Pt-tipped Au@ZIF-8 *in vivo* were recorded by infrared thermal imaging after intravenous injection (Fig. 4A). With 1064 nm laser irradiation (1 W cm^−2^), the temperature in the tumor site treated with the DOX-Pt-tipped Au@ZIF-8 nanostructures increased to 44.8 °C within 4 min, which was sufficient to cause cell damage.^56^ In contrast, the tumor treated with PBS displayed a slight temperature change. Owing to the high X-ray absorption coefficient of Au and Pt,^57^ the Pt-tipped Au@ZIF-8 could exhibit excellent CT imaging performance. As displayed in Fig. 4C, after intratumoral injection, an obviously enhanced CT signal was observed in the tumor tissue. To investigate the catalase-like ability of the nanocomposite to relieve tumor hypoxia *in vivo*, hypoxia-inducible factor (HIF)-1a immunofluorescence staining assay was employed for tumor tissue extracted from mice after 24 h post-injection. In Fig. 4B, the DOX-Pt-tipped Au@ZIF-8-treated group shows significantly weaker hypoxia green fluorescence than the control group, demonstrating that the hypoxia status of the tumor could be efficiently ameliorated by the plasmon-enhanced catalase-like ability.

### *In vivo* combination therapeutic effect of DOX-Pt-tipped Au@ZIF-8

**Fig. 5:**
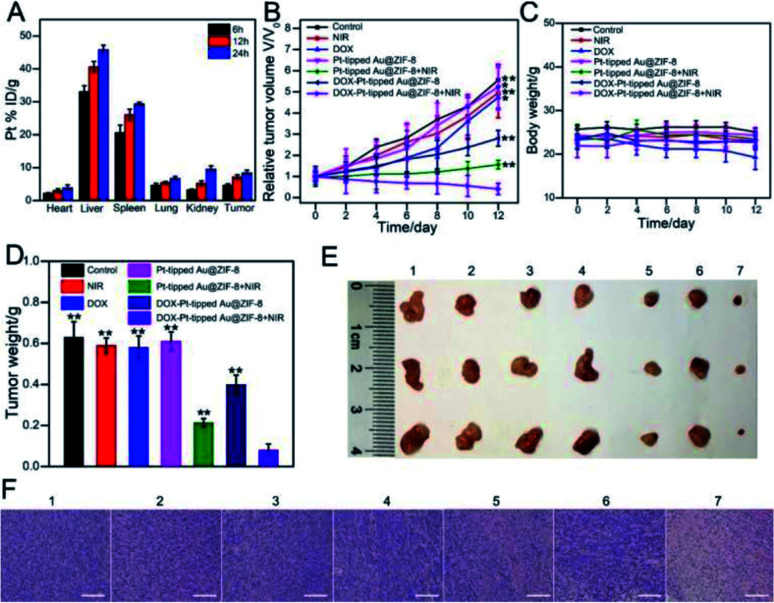
(A) The biodistribution of Pt in tumors and main organs of mice after intravenous injection with DOX-Pt-tipped Au@ZIF-8 at different time intervals. Relative tumor volume growth curves (B), body weight curves (C) and tumor weights (D) of the 4T1 tumor-bearing mice with various treatments (**p < 0.01, *p < 0.05). (E) Photographs of tumors collected from tumor-bearing mice at the end of every different treatment. (F) H&E staining images of tumor sections in each group. Group 1: PBS. Group 2: NIR-II laser irradiation. Group 3: DOX. Group 4: Pt-tipped Au@ZIF-8. Group 5: Pt-tipped Au@ZIF-8 + 1064 nm laser irradiation. Group 6: DOX-Pt-tipped Au@ZIF-8. Group 7: DOX-Pt-tipped Au@ZIF-8 + 1064 nm laser irradiation. Scale bar: 100 μm.

Then, the *in vivo* combination therapy of chemotherapy and PTT/PDT with DOX-Pt-tipped Au@ZIF-8 for mice bearing the 4T1 tumor model was studied. Firstly, the biodistribution of DOX-Pt-tipped Au@ZIF-8 was examined by measuring the Pt concentrations *via* ICP-MS in major organs and the tumor at various time points (6, 12 and 24 h) after intravenous administration. As displayed in Fig. 5A, the tumor passive-targeting efficiency of DOX-Pt-tipped Au@ZIF-8 could reach the highest accumulation amount at 24 h post-position, demonstrating effective accumulation in the tumor site *via* the enhanced permeability and retention effect (EPR)^58^ and eventually effective renal clearance.^59^ All of the 4T1 tumor-bearing nude mice were randomly divided into seven groups (*N* = 5) when the tumor volumes reached about 100 mm^3^ and received different treatments: (1) PBS, (2) only NIR laser irradiation, (3) DOX, (4) Pt-tipped Au@ZIF-8, (5) Pt-tipped Au@ZIF-8 + 1064 nm laser irradiation, (6) DOX-Pt-tipped Au@ZIF-8, (7) DOX-Pt-tipped Au@ZIF-8 + 1064 nm laser irradiation. The tumor sizes and body weight of all mice were monitored every other day after treatment. As shown in Fig. 5B, the tumors in PBS, only NIR laser irradiation, DOX and Pt-tipped Au@ZIF-8 groups grow rapidly. Compared with the DOX group, the DOX-Pt-tipped Au@ZIF-8 group displays a better tumor inhibition effect which could be attributed to the efficient specific binding between folate on the surface of the nanocomposite and overexpressed FA-receptors on the cancer cells,^60,61^ so as to enable more effective tumor enrichment. Furthermore, the tumors treated with DOX-Pt-tipped Au@ZIF-8 + 1064 nm laser irradiation combining chemotherapy and PTT/PDT were completely suppressed, better than the Pt-tipped Au@ZIF-8 + 1064 nm laser irradiation (PTT/PDT) group. From Fig. 5C, there was no significant difference in body weight in any of the test groups, except for the free DOX group, indicating the low side effect of the nanocomposites and the systemic toxicity of free DOX. After 12 days treatment, all the mice were euthanized to collect their corresponding tumors and major organs. The tumor weight (Fig. 5D), photographs of tumors (Fig. 5E) and representative tumor mice (Fig. S11†) taken at the end of different treatments also reflect the outstanding synergistic chemo-phototherapy of DOX-Pt-tipped Au@ZIF-8 under 1064 nm laser irradiation. As shown in Fig. 5F, the hematoxylin and eosin (HE) staining of the tumor tissues in each group were further investigated. There was no obvious destruction of tumor cells in the PBS, only NIR laser irradiation, free DOX and Pt-tipped Au@ZIF-8 groups. Meanwhile, the tumor treated with DOX-Pt-tipped Au@ZIF-8 with 1064 nm laser irradiation displayed massive cell death and inflammation. In addition, histological analysis of the major organs (heart, liver, spleen, lungs and kidneys) in each group was also carried out to evaluate the biosafety *in vivo* (Fig. S12†). There was no significant damage or inflammation of major organs, indicating excellent biocompatibility and a highly synergistic antitumor effect.

## Conclusions

In summary, we have successfully constructed a well-designed multifunctional nanocomposite by simultaneous encapsulation of plasmonic bimetallic Pt-tipped Au nanorods and chemotherapeutic drug DOX in a ZIF-8 matrix, which can effectively realize the synergistic chemo-phototherapy (PTT/PDT) and IR/CT imaging *in vivo* under single 1064 nm laser irradiation. Compared with the Pt fully covered Au NR nanostructures, the Pt-tipped Au NRs present more potent photothermal and photodynamic performance due to the more efficient plasmon-induced electron–hole spatial separation with 1064 nm laser irradiation. Meanwhile, nano-Pt as a nanozyme could modulate the generation of O_2_ and relieve tumor hypoxia to enhance PDT efficiency. The resulting DOX-Pt-tipped Au@ZIF-8 theranostic nanoplatform realized complete tumor growth inhibition both *in vitro* and *in vivo* with little side effect owing to the outstanding synergistic effect of combined chemo-phototherapy. This work provides an applicable strategy to integrate distinct multiple components into a stimuli-responsive “all-in-one” nanocarrier for enhanced cancer imaging diagnosis and therapy.

## Ethical statement

All animal assays obeyed the institutional animal use and care regulations approved by the Model Animal Research Center of Nanjing University (MARC). Nude mice were bought from the Model Animal Research Center of Nanjing University.

## Data availability

All experimental or computational data associated with this article have been provided in the ESI.

## Author contributions

M. C., W. Z. and J. X. conceived the idea. M. C. conducted most of the experiments. H. H. and X. Z. performed some characterization and performance tests. W. Z., H. C. and J. X. wrote the paper. All the authors discussed the results and commented on the manuscript.

## Conflicts of interest

The authors declare no competing financial interest.

## Supplementary Material

SC-012-D1SC01760H-s001
