# Supplementary material for: A plasmon-enhanced theranostic nanoplatform for synergistic chemo-phototherapy of hypoxic tumors in the NIR-II window
Source: Chem Sci. 2021 Jul 13;12(32):10848–54. doi: 10.1039/d1sc01760h (PMC8372559; doi:10.1039/d1sc01760h)
Supplement: SC-012-D1SC01760H-s001 [file SC-012-D1SC01760H-s001.pdf]

Electronic Supplementary Material (ESI) for Chemical Science.
This journal is © The Royal Society of Chemistry 2021

Supporting information

Plasmon-Enhanced Theranostic Nanoplatform for Synergistic Chemo-Phototherapy of Hypoxic Tumors in the NIR-II Window

Ming-Ming Chen[a], Hai-Li Hao[a], Wei Zhao[a]*, Xueli Zhao[b], Hong-Yuan Chen[a], Jing-Juan Xu[a]*

[a] State Key Laboratory of Analytical Chemistry for Life Science and Collaborative Innovation Center of Chemistry for Life Sciences, School of Chemistry and Chemical Engineering, Nanjing University, Nanjing 210023, PR China

[b] College of Chemistry and Molecular Engineering, Zhengzhou University, Zhengzhou 450001, China

*Corresponding Author

E-mail address: weizhao@nju.edu.cn (W. Zhao); xujj@nju.edu.cn (J.J. Xu);

Tel/Fax: +86-25-89687924

Experimental Section.

Chemicals and materials. $HAuCl_4 \cdot 4H_2O$, cetyltrimethylammonium bromide (CTAB), silver nitrate ($AgNO_3$), ascorbic acid (AA), sodium borohydride ($NaBH_4$), K_2PtCl_4 and doxorubicin hydrochloride (DOX) were purchased from Sigma-Aldrich (U.S.A). $mPEG_{2000}$-FA was purchased from 3A Chemicals Co., Ltd (Shanghai, China). Zinc nitrate hexahydrate ($Zn(NO_3)_2 \cdot 6H_2O$, 99%), 2-methylimidazole (2-MIM, 99%), polyvinylpyrrolidone (PVP, Mw = 29000), hydrochloric acid (HCl) and methanol (AR grade, 99.9%) were purchased from Sinopharm Chemical Reagent. Hoechst 33342, 1,3-diphenylisobenzofuran (DPBF), methylthiazolyldiphenyl-tetrazolium bromide (MTT), Hypoxyprobe (pimonidazole) and 2',7'-dichlorodihydrofluorescein diacetate (DCFH-DA) were purchased from KeyGEN Biotech (Nanjing, China). All chemicals were used directly without further purification.

Apparatus for characterization. Transmission electron microscopy (TEM) and high-resolution transmission electron microscopy (HRTEM) measurements were carried out on a JEOL-2100 instrument operated at 200 kV. EDX High-angle annular dark field (HAADF) STEM and STEM-EDX elemental mapping were achieved on JEM-2800 (JEOL Ltd., Japan). The X-ray diffraction (XRD) patterns were tested with a Rigaku Dmax 2500 PC diffractometer equipped with Cu Kα□ radiation (λ = 0.15405 nm). The UV-Vis absorption spectra were recorded from SHIMADZU UV-3600 spectrophotometer (Japan). Brunauer-Emmett-Teller (BET) surface area, pore volume and pore size were measured using a Quadrasorb SI-MP instrument. Confocal fluorescence images of cells were acquired with a TCS SP5 confocal microscopy (Leica, Germany). Thermal images were taken by infrared thermal camera (Fotric 225-1, Fotric, China).

Synthesis of Au NRs. Firstly, the seed solution was prepared by injecting a freshly prepared, ice-cold $NaBH_4$ solution (10 mM, 600 μL) into a mixture containing $HAuCl_4$ (10 mM, 250 μL) and CTAB (0.1 M, 9.75 mL), followed by rapid inversion for 2 min. The resultant seed solution was kept at room temperature for 2 h prior to use. Next, the growth solution for Au NRs was prepared. 2 mL of 10 mM $HAuCl_4$ was added to 40 mL of an aqueous solution containing CTAB (0.1M). Then, 0.4 mL of 15 mM $AgNO_3$, 0.8 mL of 1 M HCl and 0.32 mL of 0.1 M AA were added with gently stirring. After the solution became colorless, 200 μL of Au seed solution was added and left undisturbed at room temperature for 12 h.

Synthesis of Pt−tipped and Pt-covered Au NRs. For Pt-tipped Au NRs, 5 mL of the above Au NRs solution was added in 10 mL of an aqueous solution containing CTAB (0.1 M). 135 μL of K_2PtCl_4 (0.01 M) was added at 40 °C and left for 30 min. Finally, 270 μL of AA (0.1 M) was added at 40 °C for 12 h. The Pt-covered Au NRs were prepared by as-prepared Au NRs were centrifuged (8500 rpm, 10 min) to remove

remaining Ag^+ ions from solution and were redispersed in 0.1 M CTAB, followed by reduction of platinum.

Synthesis of DOX-Pt-tipped Au@ZIF-8. Firstly, 10 mL of the above as prepared Pt-tipped Au NRs were centrifugated at 10000 rmp for 20 min and redispersed in 10 mL of PVP (0.5g). After gently stirring for 10 h, the PVP-stabilized Au NRs were collected by centrifugation at 8,000 rpm for 30 min. The sample was redispersed in 4 mL methanol. Then, 8 mL methanol solution of 2-MIM (14.25 mg) were added and stirred for 2 min, followed by the addition of 8 mL methanol solution of $Zn(NO_3)_2 \cdot 6H_2O$ (30.382 mg). 2 h later, the final product was washed with methanol twice at 8000 rpm for 10 min. Finally, 2 mL DOX (2 mg/mL) and 2 mL mPEG-FA (2 mg/mL) were added into the Pt-tipped Au@ZIF-8 and stirred for 48 h. The resulting DOX-Pt-tipped Au@ZIF-8 were acquired by centrifugation at 8000 rpm for 10 min twice.

Photothermal performance measurement. To measure the photothermal effect of Au NRs, Pt-tipped Au and Pt-covered Au NRs, 2 mL of an aqueous suspension containing 100 μg/mL NRs were exposed to irradiation under 1064 nm laser (1 $W \cdot cm^{-2}$), respectively. The real-time temperature was recorded every 60 seconds by infrared thermal camera. The photothermal stability of Pt-tipped Au@ZIF-8 aqueous solution was measured by three cycle irradiation.

FDTD simulation. The computational simulations were performed by using the finite-difference-time-domain (FDTD) method with perfectly matched layers (PML) boundary conditions, which is performed with FDTD Solutions (Lumerical, Canada). The optical constants of Au were adopted from tabulated values for bulk gold measured by Johnson and Christy [1]. The size of the nanorod was taken to match the average value. For the model of Pt-tipped Au NR, Pt particles were located at both ends of Au NR. For the model of Pt-covered Au NR, Pt particles were homogeneously located on the surface of Au NR.

Catalase Activity Detection. To verify the catalase activity of Au NRs, Pt-tipped Au and Pt-covered Au NRs for continued production of O_2, 1 mL of an aqueous suspension containing 200 μg/mL NRs were incubated with 10 mL of H_2O_2 (30 mM). The oxygen levels were monitored over time by the dissolved oxygen meter (HI9146, HANNA instruments, Korea). Moreover, the plasmon-enhanced catalase activity of Au NRs, Pt-tipped Au and Pt-covered Au NRs were also measured with 1064 nm laser irradiation (0.5 $W \cdot cm^{-2}$) from the 5th min to the 12th min.

Detection of $^1O_2$. DPBF was selected as a probe to detect the generation of singlet oxygen. In a typical process, Pt-tipped Au NRs (100 μg/mL), H_2O_2 (1 mmol/L) and DPBF (20 μg/mL) was added into 2 mL of PBS. The DPBF absorption peak at 410 nm was recorded by UV-Vis spectrophotometer at 0, 5, 10, 15, 20, 25 and 30 min after irradiation with 1064 nm laser (0.2 $W \cdot cm^{-2}$). ESR was performed on a Bruker-E500

ESR spectrometer. TEMP was chosen as the trapping agents to detect $^1O_2$. Typically, 20 μL of TEPM and 10 μL of H_2O_2 (1 mM) were added to 100 μL PBS, Au NRs, Pt-tipped Au and Pt-covered Au NRs solutions (100 μg/mL). Then, the solutions were irradiated with 1064 nm laser (0.5 $W \cdot cm^{-2}$) for 5 min and further detected by the ESR spectrometer.

Drug loading and pH/NIR dual stimuli-responsive controlled release. 2 mL of DOX (1 mg/mL) was mixed with Pt-tipped Au@ZIF-8 (1 mg/mL, 2 mL), and the mixtures were stirred for 24 h at 25 °C under dark conditions. The precipitate was separated by centrifugation and washed several times until the supernatant was almost colorless. The drug loading efficiency (DLE %) of DOX was calculated by the below equation:

$$\text{DLE (\%)} = [m_{\text{original DOX}} - m_{\text{DOX in supernatant}}] / [m_{\text{original DOX}}] \times 100\%$$

The in vitro drug release behavior of the DOX-Pt-tipped Au@ZIF-8 was performed in PBS solutions with different pH values (pH = 7.4 and 5.2) and with or without 1064 nm laser irradiation. The amounts of released DOX in the supernatant solutions were measured by UV-Vis spectrophotometer at λ= 480 nm.

In vitro cellular uptake efficacy of DOX-Pt-tipped Au@ZIF-8. 4T1 cells were cultured in RPMI-1640 medium and grown in a cell culture incubator at constant temperature of 37°C with 5% CO_2. Cellular uptake can be investigated by intracellular DOX fluorescence. The 4T1 cells were pre-seeded in confocal dishes for 24 h. Then the cells were incubated with the DOX-Pt-tipped Au@ZIF-8 (100 μg/mL) for different times (1 h and 4 h) and stained with Hoechst 33342 for 10 min. After coculturing for 3.5 h, the 1064 nm laser (1 $W \cdot cm^{-2}$) was used to irradiate the cells. After that, the cells were incubated for another 0.5 h to image by confocal laser scanning microscopy (CLSM).

Intracellular ROS detection. DCFH-DA is a fluorescent marker for intracellular ROS, which can be oxidized by ROS and emits bright green fluorescence. Firstly, 4T1 cells were incubated at 37 °C under 5% CO_2 for 24 h and then incubated with 400 μL of Au NRs, Pt-tipped Au and Pt-covered Au NRs (100 μg/mL) for 4 h, respectively. After washing the cells with PBS, the 2', 7'-dichlorodihydrofluorescein diacetate (DCFH-DA) was added and incubated for 20 min at 37 °C. Finally, the cells were irradiated by the 1064 nm (1 $W \cdot cm^{-2}$) laser for 5 min and fluorescence intensity was measured on confocal laser scanning microscopy.

In vitro cytotoxicity measurement. Cell viability was determined by the standard MTT assay. Firstly, dose dependent cytotoxicity of the Pt-tipped Au@ZIF-8 nanocomposite against both 4T1 and Hela cells was evaluated under dark conditions. 4T1 and HeLa cells were pre-plated in 96-well plates (1×10^4 cells per

well) and cultured overnight. Then, the Pt-tipped Au@ZIF-8 dispersions with different concentrations (0, 10, 20, 50, 100, 200 μg/mL) were added and continued to incubation at 37 °C for 24 h and were detected by investigating the viability of cells via MTT assay. The cell viability of different structural nanocomposites for Au@ZIF-8, Pt-tipped Au@ZIF-8 and Pt-covered Au@ZIF-8 were also studied by the MTT assay with 1064 nm laser irradiation. Then, the in vitro cytotoxicity induced by synergistic chemotherapy/PTT/PDT was evaluated by MTT assay. Briefly, 4T1 cells were seeded in 96 well plates at a density of 1×10^4 cells per well and grown in 5% CO_2 at 37 °C overnight. Then free DOX (50 μg/mL), Pt-tipped Au@ZIF-8 (200 μg/mL), DOX-Pt-tipped Au@ZIF-8 (200 μg/mL) were added to the medium, and the cells were incubated for 4 h. The media containing samples were washed out and the photothermal and photodynamic treatment was irradiated by 1064 nm laser under 1 $W\cdot cm^2$ for 5 min. After that, the cells were incubated for 24 h. The MTT process was carried on following the same procedure.

In vitro live/dead cell staining assay. The live/dead cell staining assay was carried out on 4T1 cells using Calcein-AM/PI double stain kit. Similarly, 4T1 cells were seeded in confocal dishes and incubated with above-mentioned different materials for 4 h. After incubation, the photothermal and photodynamic therapy was subjected to NIR laser irradiation for 5 min. Then, the cells were costained with Calcein AM and PI for 20 min. After that, the cells were washed with PBS and imaged on fluorescence microscope.

In vivo multimodal imaging. All animal assays obeyed the institutional animal use and care regulations approved by the Model Animal Research Center of Nanjing University (MARC). Nude mice were bought from model animal research center of Nanjing University and the 4T1-tumor bearing nude mice were established by subcutaneous injection of 4T1 cells (1×10^6 cells). When the tumor volumes reached to about 100 mm^3, mice bearing 4T1 tumors were injected with 100 μL of DOX-Pt-tipped Au@ZIF-8 (1 mg/mL) for IR/CT imaging. Photothermal imaging was performed with the IR thermal imaging camera after intravenous injection by 1064 nm laser irradiation (1 $W\cdot cm^{-2}$). CT images were obtained from the Philips 256 CT scanning system after intratumoral injection.

Immunofluorescence of tumor hypoxia. The mice were injected with PBS (control) or the DOX-Pt-tipped Au@ZIF-8 (200 μL, 1 mg/mL) and exposed to 1064 nm laser irradiation (1 $W\cdot cm^2$) for 5 min after 24 h. Then, the mice were intravenously injected with pimonidazole hydrochloride (60 mg/kg). 30 min later, the mice were sacrificed and the tumors were surgically excised, formalin fixed, then embedded in paraffin. In order to detect pimonidazole, the tissue sections were incubated with mouse FITC-conjugated anti-pimonidazole antibody and rabbit anti-FITC rabbit secondary antibody (dilution 1:100) according to the

kit's instructions. Cell nucleis were stained with DAPI (dilution 1:2000). Images were obtained by confocal fluorescence microscopy.

In vivo biodistribution analysis. For biodistribution analysis, mice bearing 4T1 tumors were injected with DOX-Pt-tipped Au@ZIF-8 (100 μL, 20 mg/kg) intravenously. Then the mice (n = 3) were euthanized at different time points (6 h, 12 h, and 24 h). The major organs (heart, liver, spleen, lung and kidney) and tumors were collected in a beaker to be weighed. Then all of the organs and tumors were heated in concentrated nitric acid and H_2O_2 (v/v = 1:2) at 70 °C until the solution became clear. The concentrations of Pt in the solutions were measured by ICP-MS, and the concentrations in each organ and tumor were calculated.

In vivo antitumor effect analysis. For in vivo synergistic therapy, the mice were randomly divided into seven groups (n=5) and treated with: (1) PBS, (2) only 1064 nm laser, (3) DOX (100μL,5 mg/kg), (4) Pt-tipped Au@ZIF-8 (100 μL, 20 mg/kg), (5) Pt-tipped Au@ZIF-8+1064 nm laser (100 μL, 20 mg/kg), (6) DOX-Pt-tipped Au@ZIF-8 (100 μL, 20 mg/kg), (7) DOX-Pt-tipped Au@ZIF-8+1064 nm laser (100 μL, 20 mg/kg). For NIR irradiation group, at 24 h post-injection, the tumor regions were irradiated with 1064 nm laser (1 $W\cdot cm^{-2}$) for 5 min. The body weights and tumor volumes were monitored every other day. The tumor size was defined as $V=ab^2/2$, where a and b are the tumor length and width, respectively.

Histology analysis. After 12 day treatments, all mice were euthanized for collecting the tumors and major organs including the heart, liver, spleen, lung, and kidney. The tissues were fixed in 10% neutral buffered formalin overnight, embedded in paraffin, and then cut with a microtome. Next, the sections were stained with hematoxylin and eosin (H&E), and the images were obtained with an optical microscope.

Statistical analysis. Data were given as mean ± standard deviation. The *t* test was adopted to execute statistical analysis with the software SPSS. Statistical significance was denoted by an asterisk (*$p < 0.05$, **$p < 0.01$).

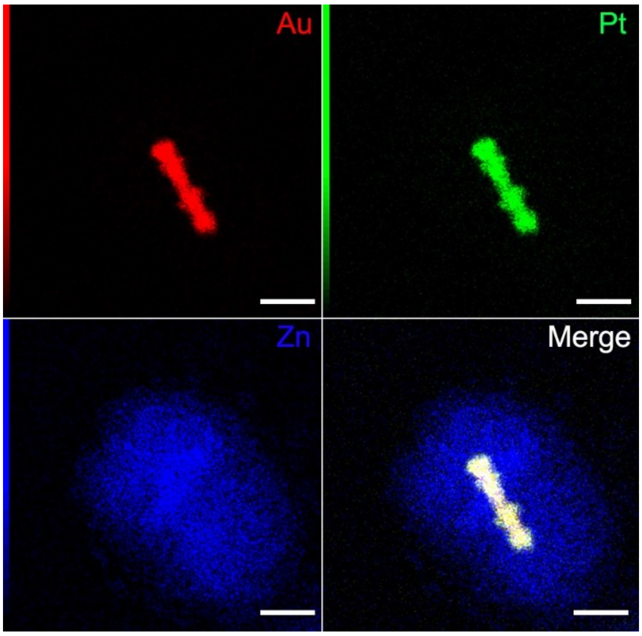

Fig. S1. STEM-EDX elemental mapping images of Au, Pt, Zn and Pt-covered Au@ZIF-8.

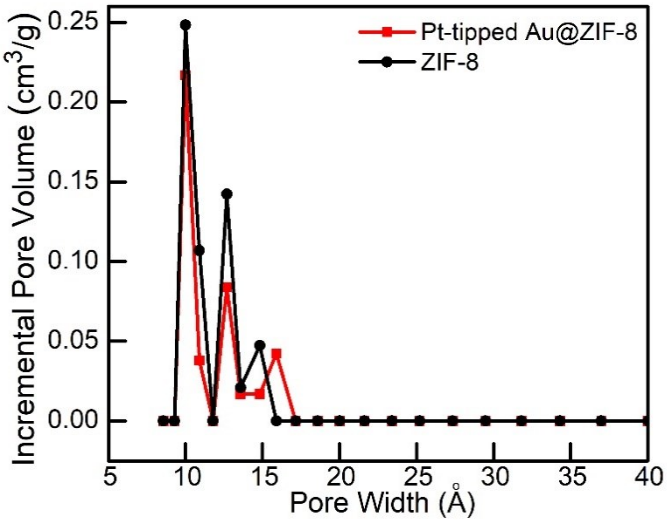

Fig. S2. The pore size distributions of pure ZIF-8 nanocrystals and Pt-tipped Au@ZIF-8 core-shell nanostructures calculated by nonlocal density functional theory.

As shown in Fig. S2, the pore size distributions of pure ZIF-8 nanocrystals and Pt-tipped Au@ZIF-8 core-shell nanostructures were calculated by nonlocal density functional theory. The pore size distribution of Pt-tipped Au@ZIF-8 is composed of three

species of micropores with diameters of 9.9, 12.7, and 15.9 Å, which were almostly consistent with that of pristine ZIF-8.

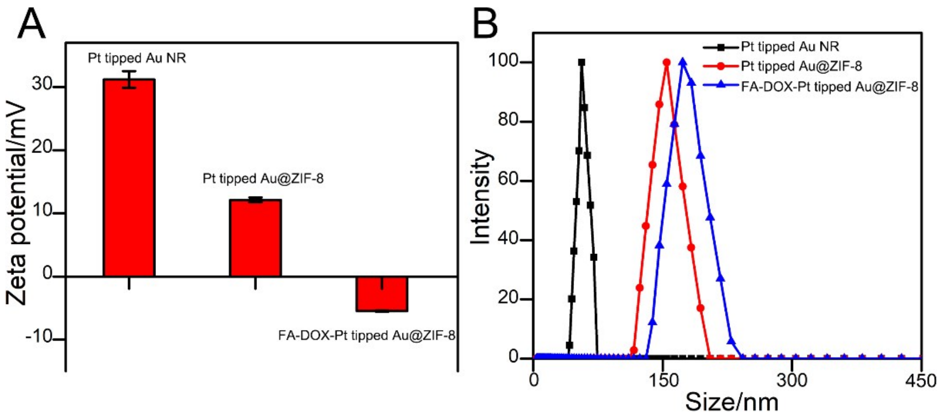

Fig. S3. Zeta potential (A) and statistical data of the diameter (B) of Pt-tipped Au NRs, Pt-tipped Au@ZIF-8 and FA-DOX-Pt-tipped Au@ZIF-8.

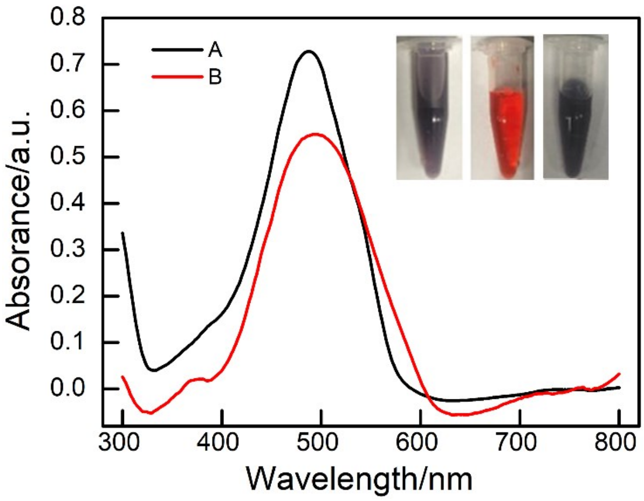

Fig. S4. UV-Vis spectra of (A) the original DOX solution and (B) the residual DOX solution after removing the Pt-tipped Au@ZIF-8 complexes. Inset: Photographs of the aqueous solutions containing Pt-tipped Au@ZIF-8, pure DOX, and DOX-Pt-tipped Au@ZIF-8 complexes, respectively.

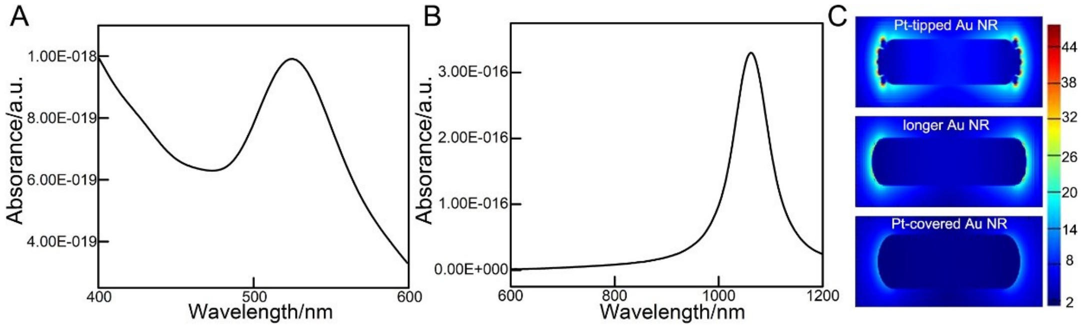

Fig. S5. FDTD simulated T-SPR (A) and L-SPR (B) of longer Au NRs with the aspect ratio of 6.2. (C) Electric field enhancement distributions of Pt-tipped Au NR, longer Au NR and Pt-covered Au NR at 1064 nm excitation based on FDTD simulation.

The Au NRs obtained were centered at about 880 nm with the average aspect ratio of 5.2. For Pt-tipped Au NRs, Pt exhibits the island growth pattern (Volmer−Wever mode) and preferentially grow on the rod tips. After tip-coating, the average length and diameter of Pt-tipped Au NRs (over 50 rods for analysis) were 58 nm and 10 nm with the average aspect ratio of 5.8. The Pt-tipped Au NRs show strong red shift to about 1060 nm in NIR-II window owing to the dumbbell-like shape and the increase of aspect ratio during deposition, in accordance with the previous report.[2] For Pt-covered Au NRs, the Au NRs were completely covered with thin Pt layer. An obvious decrease in LSPR intensity and slight red shifts was observed, which was attributed to the thin Pt layer inhibits the light absorbance. In order to further illustrate the advantages of the Pt-tipped Au NRs, we performed FDTD simulation. Taking longer Au NRs with the average length and diameter of 62 nm and 10 nm and aspect ratio of 6.2, the LSPR absorption spectrum could be centered at about 1064 nm (Fig.S5B). However, the electric field enhancement of longer Au NRs under 1064 nm laser excitation is still lower than that of the Pt-tipped Au NRs (Fig. S5C).

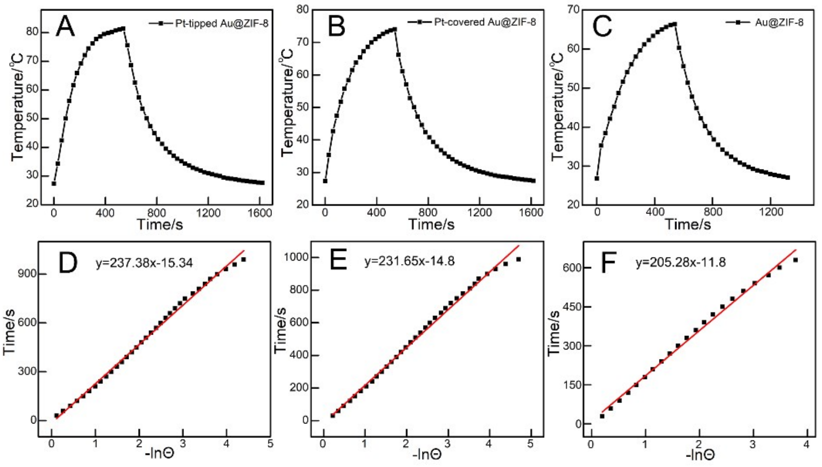

Fig. S6. Temperature change curves of Pt-tipped Au@ZIF-8 (A), Pt-covered Au NR@ZIF-8 (B) and Au@ZIF-8 (C) with 1064 nm laser irradiation. Time constant for heat transfer from the Pt-tipped Au@ZIF-8 (D), Pt-covered Au@ZIF-8 (E) and Au NR@ZIF-8 (F) solution irradiated with 1064 nm laser.

Calculation of Photothermal Conversion Efficiency (*η*): The photothermal conversion efficiency is calculated by formula (1)[3]:

$$\eta = \frac{hS(T_{max,NP} - T_{surr}) - Q_{dis}}{I(1 - 10^{-A_{1064}})} \qquad (1)$$

where h is the heat transfer coefficient, S is the surface area of the container, $T_{max,NP}$ is the maximum temperature of the solution (obtained from Figure S6), T_{surr} is the surrounding temperature (27.4 °C, obtained from Figure S6), I is the laser power density (1 W·cm^{-2}), A_{1064} is the absorption value (obtained from Figure 2A) of the material at 1064 nm, and Q_{dis} is the heat generated after water and container absorbs light. To calculate hS, equations (2) and (3) were introduced:

$$Q_{dis} = hS(T_{max,H_2O} - T_{surr}) \qquad (2)$$

$$\tau_s = \frac{m_D C_D}{hS} \qquad (3)$$

$T_{max,H2O}$ is the maximum temperature of the water (34.1 °C, obtained from Figure 2B, black curve), m_D is the mass of water (0.5 g), C_D is the heat capacity of water (4.2 J·g^{-1}·°C^{-1}), and τ_s is the sample system time constant, which was calculated by formula (4) (5):

$$t = -\tau_s ln\theta \tag{4}$$

$$\theta = \frac{T_{surr} - T}{T_{surr} - T_{max}} \tag{5}$$

From the slope of fitting curve in Figure S6, the sample system time constant τ_s were found to be 237.38 (Pt-tipped Au@ZIF-8), 231.65 (Pt-covered Au@ZIF-8) and 205.28 (Au NR@ZIF-8). Thus, according to calculating, the heat conversion efficiency (η) of the sample under 1064 nm laser is about 42.1% (Pt-tipped Au@ZIF-8), 40.2% (Pt-covered Au@ZIF-8) and 39.2% (Au NR@ZIF-8).

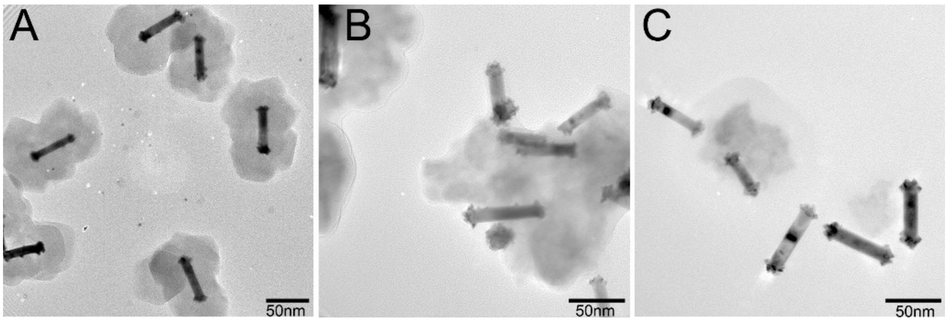

Fig. S7. TEM images of (A) DOX-Pt-tipped Au@ZIF-8 at pH 7.4 after 6h, (B) DOX-Pt-tipped Au@ZIF-8 at pH 5.2 after 6h and (C) DOX-Pt-tipped Au@ZIF-8 at pH 5.2 with 1064 nm laser irradiation after 6h.

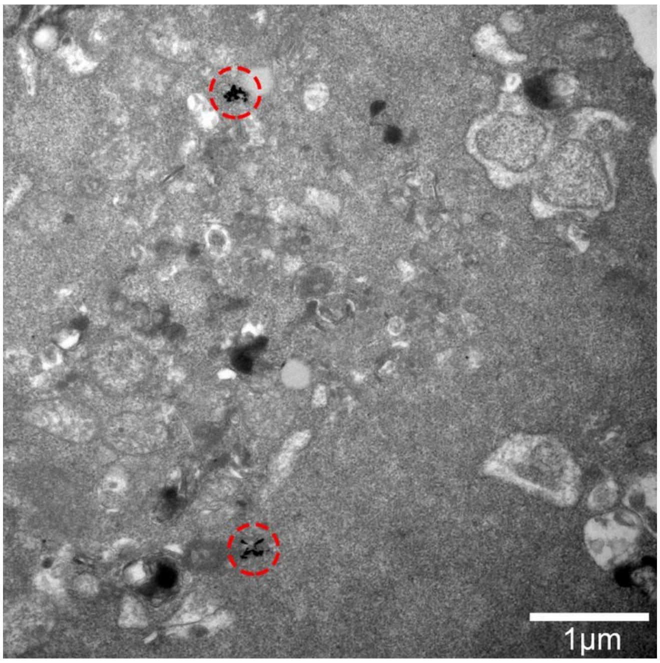

Fig. S8. Cell TEM images of fine slices of 4T1 cell incubated with the DOX-Pt-tipped

Au@ZIF-8.

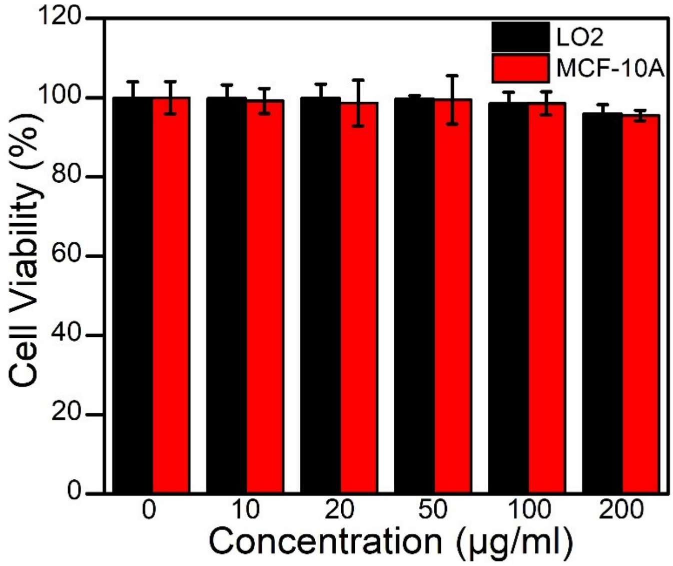

Fig. S9. MTT viability assessment of normal LO2 and MCF-10A cells treated with different concentrations of Pt-tipped Au NRs.

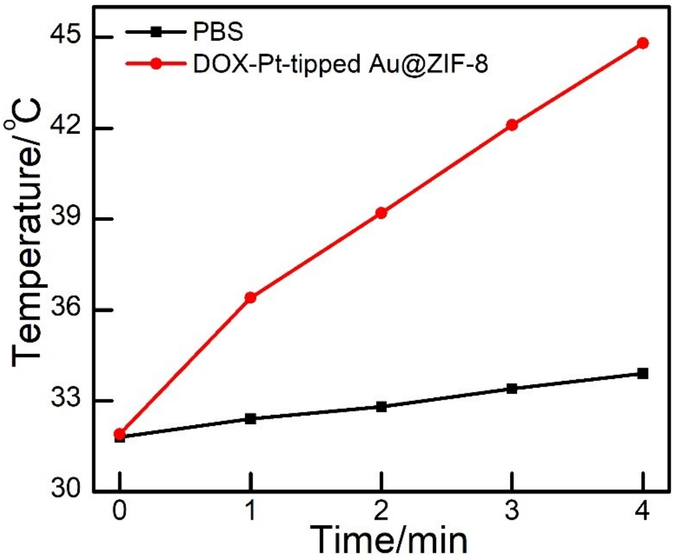

Fig. S10. The quantification and statistical analysis of tumor temperature treated with PBS and DOX-Pt-tipped Au@ZIF-8 under NIR-II laser

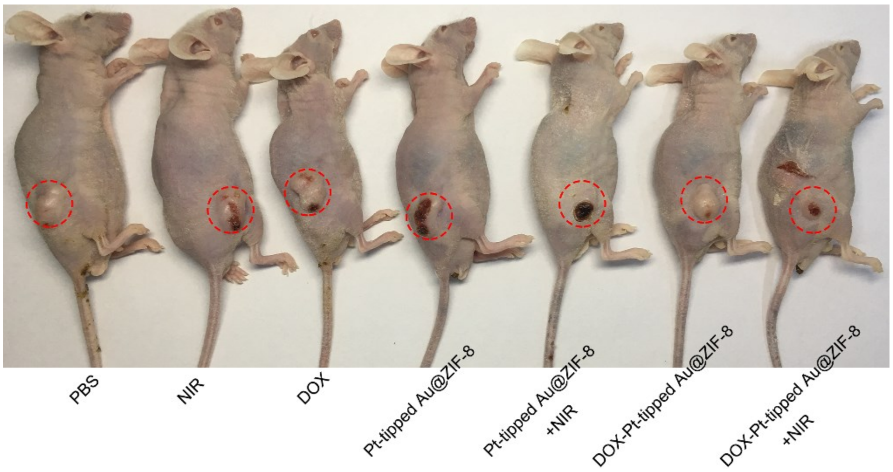

Fig. S11. The representative photographs of tumor-bearing mice with different treatments after 12 days.

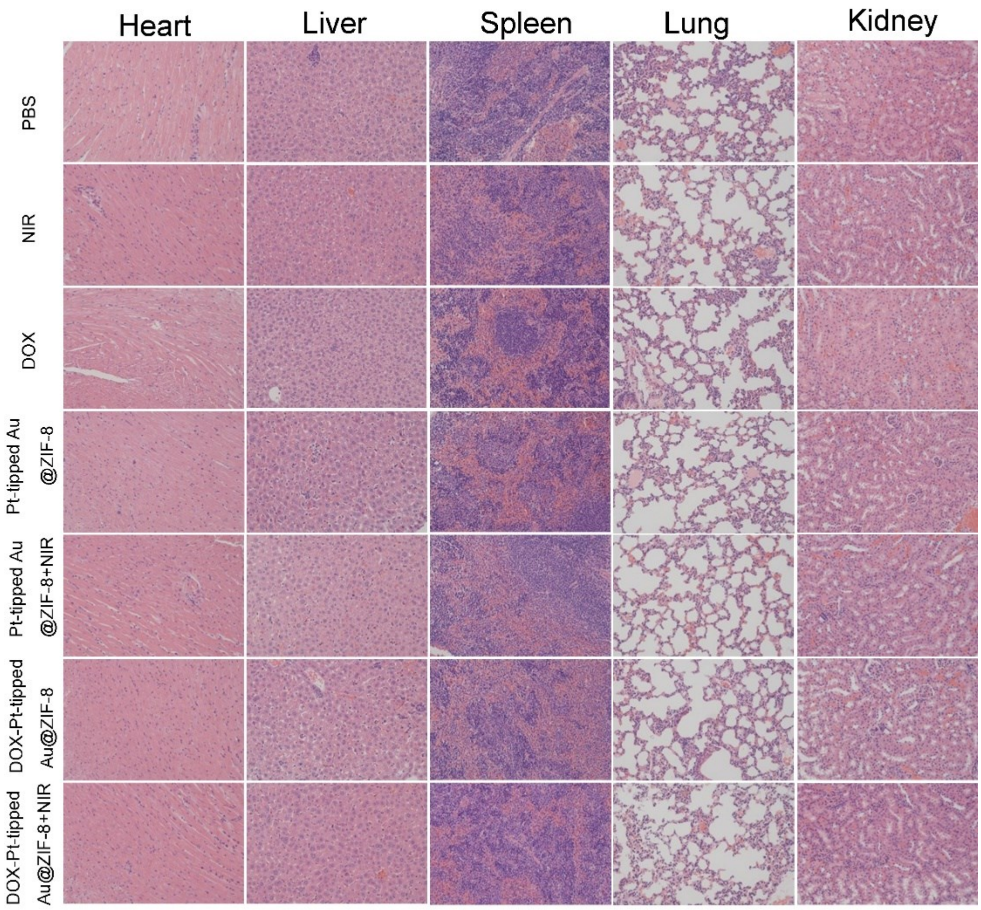

Fig. S12. H&E staining tissue sections of major organs harvested from mice in different groups.

References

1. P. B. Johnson and R. W. Christy, Phys. Rev. B, 1972, 6, 4370-4379.
2. M. Grzelczak, J. Pérez-Juste, F. J. García de Abajo and L. M. Liz-Marzán, J. Phys. Chem. C, 2007, 111, 6183-6188.
3. H. Chen, L. Shao, T. Ming, Z. Sun, C. Zhao, B. Yang and J. Wang, Small, 2010, 6, 2272-2280.
